# A New Solid Solution Approach for the Study of Self-Irradiating Damage in non-Radioactive Materials

**DOI:** 10.1038/s41598-017-03150-9

**Published:** 2017-06-05

**Authors:** Tzvi Templeman, Michael Shandalov, Michael Schmidt, Amir Tal, Gabby Sarusi, Eyal Yahel, Itzhak Kelson, Yuval Golan

**Affiliations:** 1 Department of Materials Engineering, Ben-Gurion University of the Negev, Beer Sheva, 84105 Israel; 2 The Ilse Katz Institute for Nanoscale Science and Technology, Ben-Gurion University, Beer Sheva, 84105 Israel; 3 Department of Physics, Nuclear Research Center Negev, P.O. Box 9001, Beer Sheva, Israel; 4 School of Physics and Astronomy, Tel-Aviv University, Tel-Aviv, 6997801 Israel; 5 Department of Electro Optics Engineering, Ben-Gurion University of the Negev, Beer Sheva, 84105 Israel

## Abstract

A new method to produce a model system for the study of radiation damage in non-radioactive materials is presented. The method is based on homogenously dissolving minute amounts of ^228^Th ions in thin films in a controllable manner using a small volume chemical bath deposition technique. This approach is demonstrated for PbS films. The properties of the PbS (^228^Th) solid solution film activity were investigated by monitoring the accompanying radioactive processes. Electrical resistivity studies were performed and decay-event damage accumulation was measured, followed by isochronal annealing which presented two annealing stages and another two sub-stages. This is the first report on self-irradiating damage studies in IV-VI semiconductors and the resulting films present a novel method for the analysis of dilute defect systems in semiconductor thin films.

## Introduction

The study of radiation damage in solid systems is of great importance for understanding the degradation of material properties in irradiating environments such as space, technology, fission and fusion reactor structural components and for nuclear waste disposal issues^1–4^. Another important aspect is the study of dilute defect systems in solids, which allows to advance understanding of material behavior under various extreme conditions and under processes similar to self-irradiation^5–8^. In the 1960’s studies have used innovative systems where the method was basically freezing noble gases containing dilute amounts of radioactive isotopes^5^. Those methods, although allowing the control over defect dose and distribution, are complex in terms of sample handling and characterization and are consequently limited to resistivity and absorption measurements. Since then, extensive studies were performed on actinide-host phases containing short lived actinides such as ^238^Pu and ^244^Cm^9, 10^. Hobbs *et al*.^9^ compared several techniques for the study of alpha-decay damage accumulation in ceramics. The specific focus was with regards to nuclear waste disposal, as such, extremely high damage rates were used and sequences leading to critical failure were monitored (swelling, cracking and amorphization). The complexity of these systems results from imposed handling restrictions due to their highly hazardous nature, which in turn limits the analytical methods through which they can be characterized. An example of handling restriction complications was presented by Booth *et al*.^11^ in their study on self-irradiation damage in Pu and Pu intermetallic alloys, clearly stating that due to handling restrictions sample ages could not be reproduced. In their study the commonly used methods for assessing damage formation and annihilation mechanisms through damage accumulation and isochronal recovery measurements were performed by monitoring changes to the local lattice structure using the EXAFS technique. Another common method uses high energy (~GeV) irradiation with ions^12–14^. These methods allow specific control over dose but are limited in use due to the inhomogeneous distribution of the defects produced and the inherent requirement for high energy accelerators. Lattice defects resulting from irradiation are mainly due to primary knock-on atoms (PKA). For alpha emitting atoms the decay results in an α-particle and a recoiling daughter atom. The α-particle having energy of ~5 MeV, loses most of its energy by excitation of lattice valence electrons. In contrast, the recoiling atom having an energy of ~0.1 MeV produces local lattice damage by creating Frenkel pair defects^1, 9, 13^. A specific method for implementing this damage process was recently reported^15^. In this method, a source of ^224^Ra (*τ*_1/2_ ~3.7 days) is placed in proximity to the thin film under study. ^220^Rn (*τ*_1/2_ ~1 minute) atoms resulting from the ^224^Ra decay are implanted into the film and initiate a chain of alpha decays, all of which contribute to the accumulating damage in the film. While this method is simple to apply and does not require complex facilities (e.g., accelerators) it suffers from some inherent shortcomings. The incurred damage depth is limited by the typical range of the recoiling atoms in the film, thus restricting study to very thin films (ca. 30 nm). In addition, the damage to the lattice is not homogenously distributed because of the geometrical asymmetry of the irradiation setup. The need for removing the film from the external ^224^Ra source for further measurements implies that the system lifetime is limited by the lifetime of ^224^Ra and is thus restricting the time available for the experiment.

**Figure 1:**
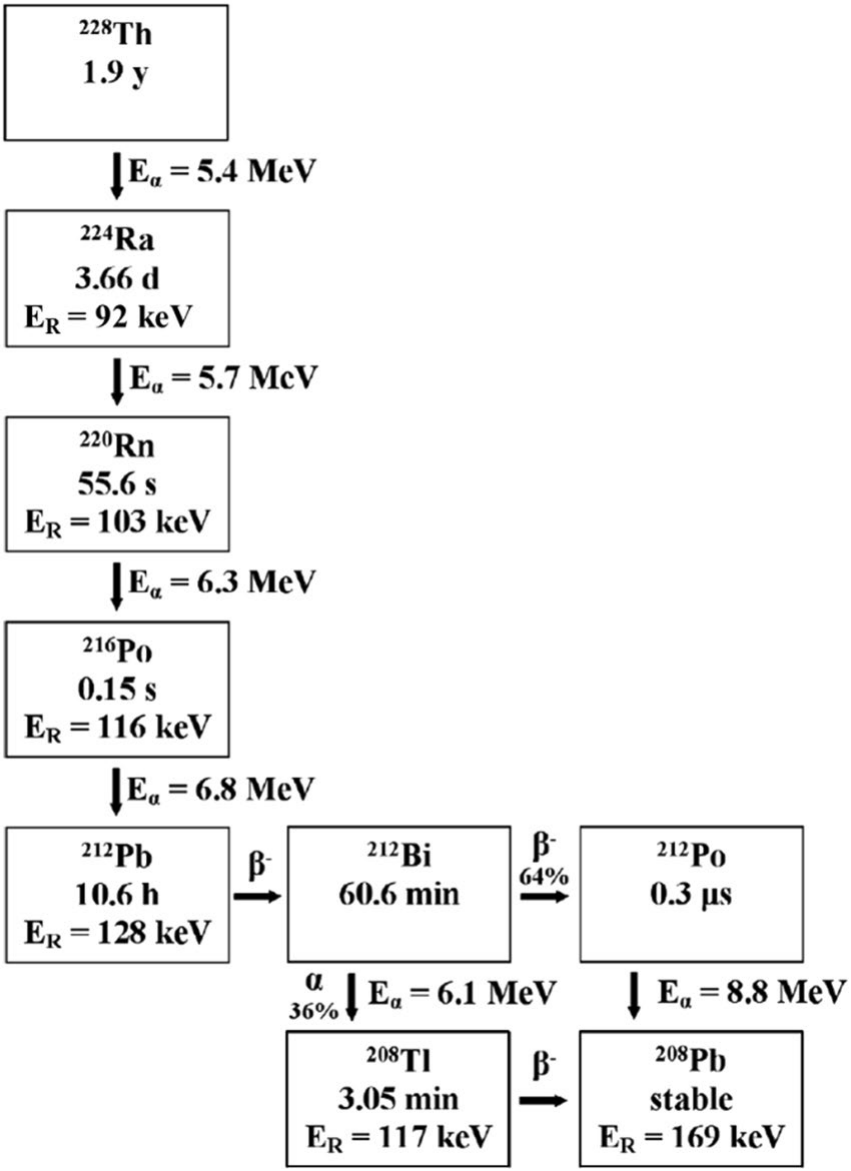
The nuclear decay chain of ^228^Th. Decay mode, daughter nuclei and recoil energy (E_R_), α-emission energy (E_α_) and the half-life are specified^45^.

The novel method which is reported in the present manuscript aims to overcome these shortcomings and to create a new radiation damage studies approach in selected systems by incorporating the ^228^Th isotopes in the thin film itself. Due to the high specific activity of ^228^Th, only very small amounts of it need to be introduced into the film to create the damage. The procedure we developed to achieve this goal takes advantage of the chemical similarity of the quasi-stable ^232^Th (1.4 × 10^10^ years half-life), for which there is no quantitative limitation. Taking advantage of the wide experience of our group in chemical bath deposition (CBD) of PbS thin films^16–20^, we have recently reported on a new method for alloying chemical bath deposited (CBD) PbS thin films by forming a solid solution with ^232^Th^21, 22^. Although PbS differs from the commonly studied metal oxides, it holds several advantages as a model system; The narrow and direct band gap establishes a new class of physical properties for damage studies, such as monitoring formation and annihilation of discrete defect-correlated energy levels in the forbidden gap. Another advantage lies in the simple rocksalt crystal structure of PbS, simplifying lattice potential calculations and damage formation simulations. A third consideration is the Th decay chain, which ends with Pb (Fig. 1), namely no foreign atoms should be present in the material at the end of the decay process.

Polyatomic ionic crystals differ significantly from metals in terms of point defect formation and recovery mechanisms. Each atom in the system presents different displacement probabilities, thus complicating the dynamics of a decay event. Moreover, the electrostatic charges which are inherent to these systems may affect point defect stability and formation probability. As an example, in ionic systems Frenkel pair defects are less probable of remaining separate due to long range columbic forces^23^. Another point to consider is the probability of lattice amorphization as a result of damage cascades. Tranchenko *et al*.^24, 25^ has extensively collected experimental data on 116 materials in an attempt to find the guiding line to predict whether a material is likely to undergo amorphization under irradiation. He discovered that the degree of ionicity between adjacent atoms is the key contributor, and the more ionic in nature, the higher the resistance to amorphization will be (higher exposure doses are required). However, electronegativity in itself is not enough to determine whether a material will be subject to amorphization, and potential distribution maps are required in order to examine charge distribution and gain insight to ionic bond nature. This preliminary requirement was performed for lead chalcogenides and it would appear that due to s-p hybridization the atomic bonds are covalent in nature, thus highly subject to amorphization^26^.

Although radiation damage in semiconducting materials has been extensively studied by external irradiation we found no reports for radiation damage in IV-VI semiconductors upon both external and internal irradiation. The absence of prior knowledge complicates analysis of defect recovery stages, but our primary goal here is to present a new method for the study of defect production resulting from self-irradiation, hence, initial assessment on categorizing defect species was handled qualitatively only. In the current work, we present first evidence for the successful replacement of ^232^Th by ^228^Th, which allow the formation of a model thin film system with a homogeneously distributed internal irradiation source. This, in turn, should function similarly to systems exposed to self-irradiation processes.

## Results and Discussion

**Figure 2:**
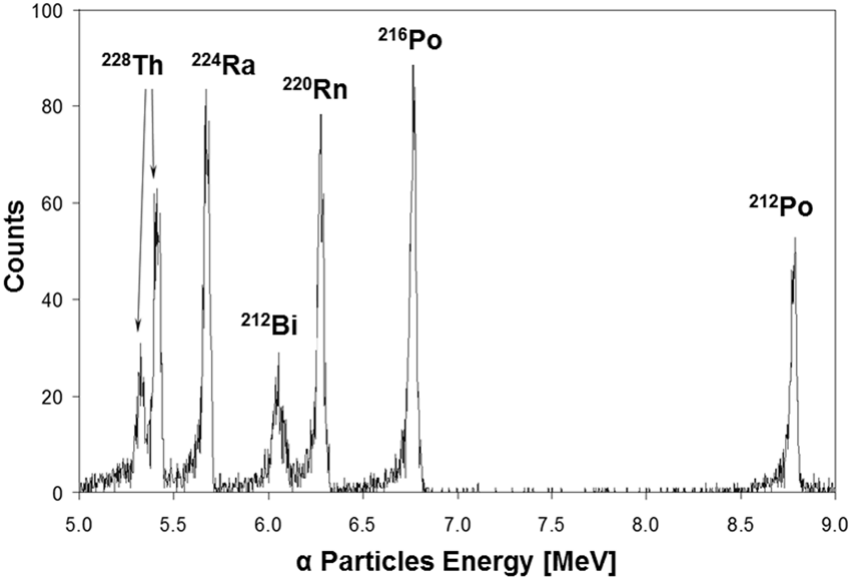
α-spectrum of the PbS (^228^Th) sample grown at pH 12.8. Acquisition duration was 6 hours, at a distance of 16 mm from the sample.

**Figure 3:**
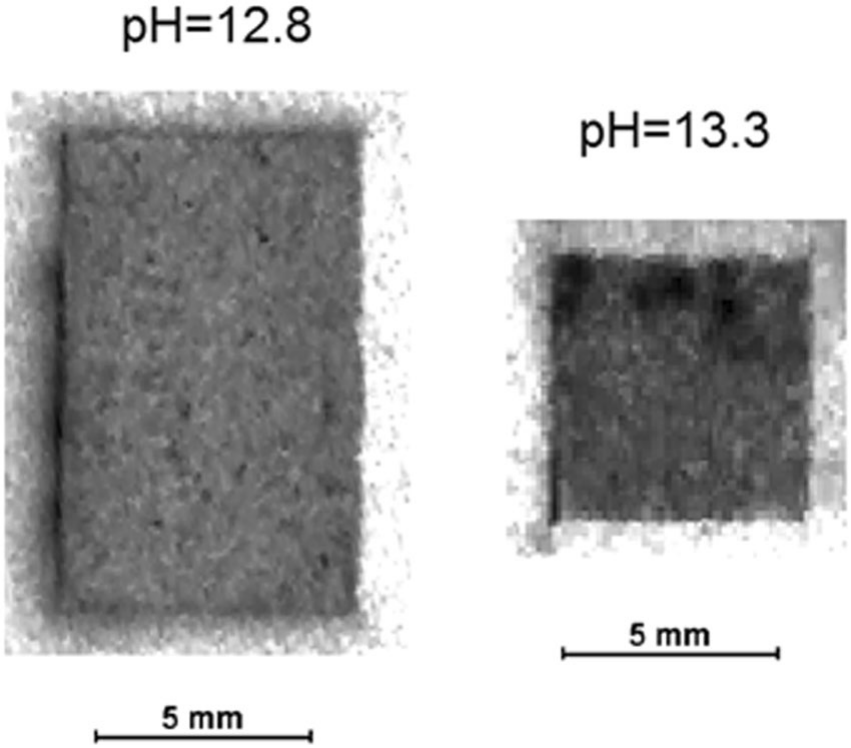
Autoradiography imaging of the PbS (^228^Th) sample grown on GaAs (100) at pH 12.8 and pH 13.3.

CBD of PbS(^232^Th) has been investigated previously by our group and homogenous distribution of incorporated ^232^Th was reported^21, 22^. In order to employ this growth method for the doping of PbS films with radioactive ^228^Th, an additional step was needed, i.e., CBD process optimization using small volume solutions. Process optimization was carried out using a minute amount of deposition solution (2 ml compared to the previous 40 ml solution) containing ^232^Th. Once stable conditions for growth were achieved using ^232^Th, the procedure was replicated using the radioactive ^228^Th isotope, although with severely reduced concentrations, to minimize hazardous waste disposal and expenses originating from the use of radioactive ^228^Th. We importantly note that while replacing ^232^Th with its radioactive isotope, we naturally include in the growth process its daughters. The chemistry of incorporating these isotopes to the film has not been studied yet (except for lead, at the end of the decay-chain, Fig. 1). With regard to thorium, we assume that the same lattice sites occupied by the non-radioactive ^232^Th isotopes are replacedby ^228^Th. By varying deposition parameters, a simple method for controlling total activity in the films was achieved. Several films were grown in this method with varying ^228^Th concentrations in solution and several emission properties were analyzed under our previous knowledge that similar morphologies were achieved for the radioactive PbS (^228^Th) and non-radioactive PbS (^232^Th) films. Emission characterization method and results are presented (Figs 2 and 3) for two samples denoted pH 13.3 and pH 12.8. Note, however, that all samples were analyzed in this method in order to verify film homogeneity and activity.

The γ-spectroscopy measurement was performed around energy line of 240 keV for both samples, i.e., growth performed at pH 13.3 (pH 12.8), where the signal comprised two lines: 241 keV (4.1%) of ^224^Ra and 239 keV (43.6%) of ^212^Pb respectively, in both samples. The total ^228^Th activity was estimated at 12 ± 1 Bq (3 ± 0.2 Bq) for pH 13.3 (pH 12.8). The low activity of the samples required performing α-spectroscopy measurements with the samples close to the detector in order to increase counting efficiency. For a detector–sample distance of 16 mm a reasonable efficiency (4%) was obtained. However, this resulted in some line broadening and a somewhat lower overall accuracy of the measurement. The resulting α-spectrum for the pH 12.8 sample was collected for 6 hours and is presented in Fig. 2. The ^228^Th total activity of samples grown at pH 13.3 (pH 12.8) was 12 ± 2 Bq (1.9 ± 0.3 Bq). Multiplying the ^228^Th activity by its lifetime and dividing by the approximate sample volume, yields an approximate ^228^Th ion concentration of 4.3 ± 0.7 · 10^13^[cm^−3^] (7.2 ± 1.1 · 10^13^[cm^−3^]) for pH 13.3 (pH 12.8). In our α-spectroscopy system a shift of one channel to lower energy corresponds to 15 nm α-passage in PbS. A shift of approximately 5 (2) channels was observed indicating an average ^228^Th depth of 150 ± 15 nm (30 ± 15 nm) and a total film thickness of 300 ± 30 nm (60 ± 30 nm) for pH 13.3 (pH 12.8) samples. This is in reasonable agreement with the thickness measurements obtained from cross-section SEM imaging (Figure S1). An independent estimate of the overall film thickness, assuming homogeneous depth distribution of ^228^Th, was obtained by measuring the ^224^Ra desorption probability from the pH 12.8 sample. This entity, which can be measured with relatively high accuracy, was 20 ± 1%, which is consistent with an average ^228^Th depth of about 30 nm.

Using the Fuji autoradiography, the samples were scanned at a resolution of 100 × 100 micrometers, which is commensurate with the range of the α-particles in PbS. From this scan we learn that ^228^Th activity is homogenously distributed over the entire samples (Fig. 3). The higher signal at the edges (apart from broken edges of the samples) is caused by the thorium precipitated on the substrate edges during film growth. Previous work established the homogenous incorporation of ^232^Th in PbS films grown using CBD and was verified, among other analytical techniques using EDS in the analytical TEM^21, 22^. While there are fundamental differences between the samples (size, geometry and composition) as well as the analytical techniques presented, all the results point out that a homogeneous distribution of Th in the films was achieved. The basic mechanisms accompanying doping of both isotopes during growth remain the same. Thus, the homogeneous incorporation of ^228^Th in our case is not surprising and other film properties are expected to be similar as well.

**Figure 4:**
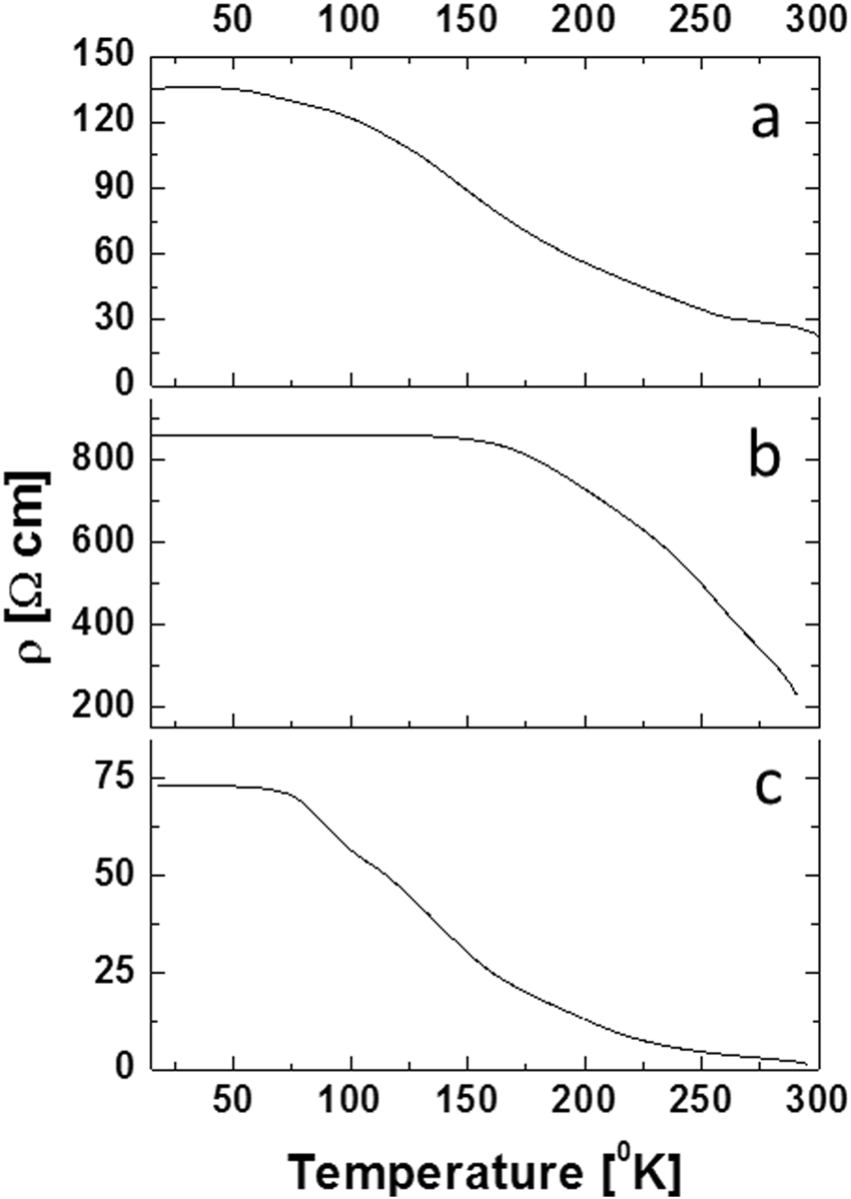
Temperature dependent resistivity measurements from room temperature to 12 °K on the various PbS films: (**a**) 0.2 at% ^232^Th doping. (**b**) 0.15 ppm ^228^Th doping accumulating damage for 6 months at room temperature. (**c**) 0.15 ppm ^228^Th doping after thermal annealing at 548 °K for 3 hr.

By implementing this method, several films were uniformly doped with the radioactive ^228^Th isotope in a controllable manner with concentrations ranging between 0.15 ppb up to 0.15 ppm. Films presenting a high activity of 65 Bq (0.15 ppm ^228^Th) were chosen for the study of self-irradiating damage processes. Temperature dependent four-probe resistivity measurements were performed between 300 and 12 °K in a radiation shielded vacuum system on non-radioactive PbS(^232^Th) films at varying doping levels. This was done in order to establish base values for the characterization of damage processes in the radioactive films. A negative temperature coefficient was observed in the films, $\frac{d\rho }{dT} < 0$, as seen in Fig. 4. These results differ significantly from literature reports on lead chalcogenides, where a positive coefficient is expected due to the mobility dependence on temperature. While text-book behaviour of semiconductors predicts *μ* ∝ *T* ^3/2^ due to ionized impurity scattering of charge carriers dominating at low temperatures, lead chalcogenides present an unusually high static permittivity, resulting in negligable scattering due to ionized impurities at low temperatures^27^. This explains both the uncommonly high mobility values at low temperatures (below 50 °K) and the decreasing behavior up to room temperature resulting in the commonly reported positive temperature coefficient of the resistivity in both single and polycrystalline lead chalcogenides. Another characteristic of lead chalcogenides is a constant carrier concentration below 300 °K, which is commonly reported independently of material microstructure, indicating that variations in resistivity values are directly controlled by mobility of charge carriers^27–29^. It appears in the present case that an activated temperature dependency exists, as such, ionized impurity scattering does not account for the resistivity behavior, and a different mechanism is dominant. This abnormal behavior was previously reported in nanocrystalline lead chalcogenides, with an average grain size under 400 nm^30–32^. It was argued that grain boundary potential barrier scattering is the dominant scattering mechanism at low temperatures, therefore, above a certain temperature sufficient kinetic energy results in an abrupt increase in mobility. The average film grain size is ~150 nm (Figure S2), thus, it is quite feasible that grain boundaries acting as trapping centers for charge carriers are dominating the scattering processes. Table 1 presents the various film properties, including activation energy values which were extracted by fitting ln(T^1/2^/ρ) as a function of 1/T. Although films contain different isotope concentrations, they present similar grain size (Figure S2), so that comparing film properties in this case is relevant. Room temperature resistivity values for ^232^Th doped nanocrystalline films are in agreement with reports on nanocrystalline PbS films (~30 [Ω · cm])^33^. Energy barrier values are also in agreement with reported values for lead chalcogenides^30, 31, 34^.

**Table 1:** Sample resistivities and extracted barrier energies (E_B_).

Description	ρ_12K_ [Ω · cm]	ρ_300K_ [Ω · cm]	ρ_300K_/ρ_12K_	E_B_ [meV]
PbS(0.2at% ^232^Th)	135.57 ± 0.05	22.69 ± 0.04	1/6	37 ± 1
PbS(0.15 ppm ^228^Th) post annealing	73.34 ± 0.03	1.28 ± 0.05	1/57	52 ± 1
PbS(0.15 ppm ^228^Th) pre annealing	859.81 ± 0.06	231.3 ± 0.1	1/4	331 ± 3

**Figure 5:**
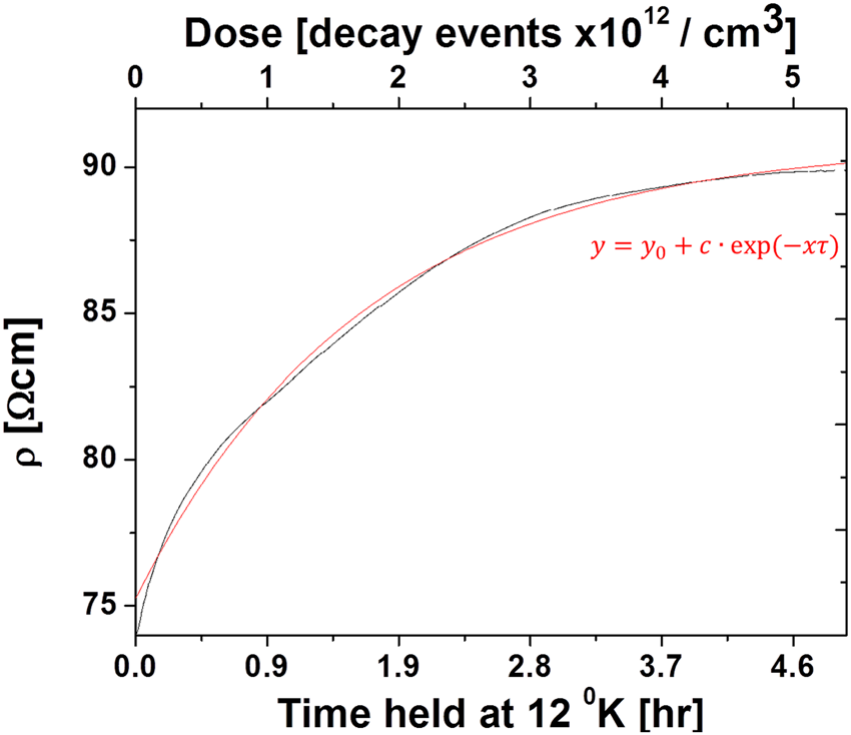
Damage accumulation in a PbS film doped with 0.15 ppm ^228^Th after thermal annealing at 548 °K for 3 hr. Decay events were calculated for the entire decay chain.

**Figure 6:**
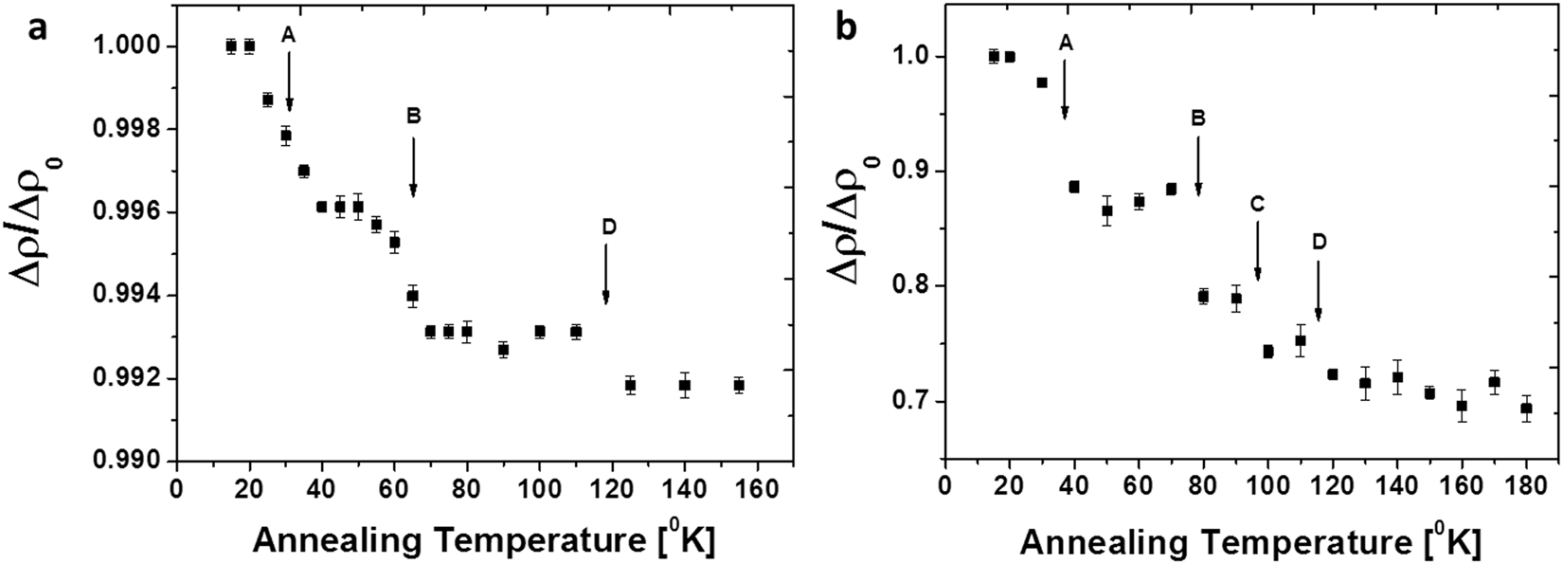
Isochronal annealing measurements (10 min annealing steps). (**a**) Pre-annealing. (**b**) Post-thermal treatment at 548 °K for 3 hr.

The study of self-irradiation damage processes was performed on PbS (0.15 ppm ^228^Th) films, samples accumulating damage for ~6 months at room temperature (pre annealing) and samples annealed for 3 hours at 548 °K in an inert atmosphere (post annealing) were analyzed. Damage accumulation measurements were performed at a constant temperature of 12 °K by acquiring resistivity as a function of time, assuming the temperature is below the first annealing stage. Defect formation resulting from decay events are expected to directly affect the resistivity as residual electrical resistivity is porportional to the sum-total radiation induced point defects, according to: ${\rm{\Delta }}\rho =\sum _{i}{c}^{i}{\rho }_{f}^{i}$ (where *c* refers to defect concnetration of type (*i*) and *ρ* is the relative resistivity contribution)^23^. No damage accumulation was observed in the pre-annealed sample over a period of 60 hours. Considering the extremely high resistivity and E_B_ values (Table 1), we assume that saturation in damage accumulation has already occurred, i.e., additional decay events in already damaged regions will not result in further resistivity increase^11, 15, 35^. Post annealing damage accumulation is presented in Fig. 5. Resistivity increases according to a single exponential component, indicating a single dominant damage formation mechanism. Considering the system at hand, the likely dominant mechanism is the formation of Schottky pair defects and over long durations, lattice amorphisation. However, without prior knowledge on displacement cross sections and resistivity change per defect, quantitative analysis is challenging. Isochronal annealing measurements were performed on both pre- and post-annealing samples and are presented in Fig. 6. Measuring Δ*ρ* as a function of temperature is one of the most common methods for studying defect reaction kinetics as it isolates between different variants of defects. Two annealing stages with sub-stages are observed in the measured temperature range. Pronounced steps are observed between 20–40 °K and 50–80 °K for both pre- and post-annealed films with additional smaller steps at 90 °K (not observed prior to annealing) and 110 °K. In FCC metals, increasing the annealing temperature induces the initial recovery steps according to the following sequence: recombination of correlated defects, uncorrelated defects and migration of di-interstitials^36^. Pure FCC metals, such as Ag, Cu, Ni and Al present stage I recovery at ~40 °K with slight variations in peak location which increases according to the lattice cohesive strength; in all cases this stage is associated with Frenkel pair defects^37^. Polyatomic ionic systems present a more complex situation where decay events may result in local structure amorphization, Schottky dimers and pairs, both cation and anion Frenkel pairs as well as vacancies and interstitials. Thus, the initial recovery stages should likely be categorized differently than in metals. Several reports on radiation damage in GaAs identified stage I recovery at 20 °K, which was attributed to As Schottky pairs^38, 39^. Contrary to these findings in InSb stage I was catogarised at 80 °K and associated with annihlation of Schottky dimers^40^. No prior data exist for the current PbS system, however, first principle studies on point defect formation energies in lead chalcogenides were considered for the analysis of defect recovery stages^41^. Considering that for most systems, formation energy will be proportional to defect migration energies, it would appear that recovery stages will initiate with correlated Schottky dimers followed by uncorrelated Schottky pairs, cation vacancies and lastly anion vacancies. Annihilation of Frenkel pairs and interstitial migration would appear less favorable in the lead chalcogenide systems. Table 2 summarizes observed isochronal steps in pre and post annealed samples. A pronounced difference between the samples can be observed in the recovery fraction. Prior to annealing, damage saturation has already occurred, due to a long period of accumulation at room temperature. This can be observed through the high resistivity values (Table 1) and the absence of damage accumulation at 12 °K. Moreover, examining the recovery fractions during annealing steps (Table 2) presents low values of under ~1% in the measured range. Once thermal annealing is performed, step recovery increases by ~33%. This behavior points to the formation of defects during decay events which are not annealed below room temperature and are dominating the scattering mechanisms. As discussed above regarding charged grain boundaries, we can assume that decay events produce local amorphization, most of which is annealed immediately through thermal spikes; however it would appear that a small percentage endures and damage accumulation proceeds slowly even at room temperature.

**Table 2:** Isochronal recovery steps temperature and recovery fraction.

Observed steps of isochronal annealing		A	B	C	D
Pre Annealing	Temperature[°K]	29 ± 20	63 ± 15	—	120 ± 40
	Recovery fraction [%]	0.4	0.3	—	0.1
Post Annealing	Temperature[°K]	35 ± 20	77 ± 15	95 ± 5	115 ± 5
	Recovery fraction [%]	13.5	8.5	5	6

## Conclusions

Chemical bath deposition was applied to create a simple, controllable and cost-effective procedure for doping PbS thin films with radioactive thorium, thus, establishing a dilute system specially designed for the study of radiation damage in semiconductor thin films. ^228^Th isotopes were successfully incorporated into PbS films using small volume CBD, a procedure which is critical when using radioactive isotopes. Homogenous incorporation of ^228^Th isotopes throughout the film was observed. α- and γ- spectroscopy measurements were performed on the PbS (^228^Th) films. Total activity, film thickness and homogenous distribution of the ^228^Th isotopes (film depth and in-film plane) were presented. Control over the concentration of Th isotopes was demonstrated, an important factor that determines defect density and distribution. Electrical resistivity measurements were performed to monitor damage accumulation followed by isochronal annealing to assess initial recovery stages. Planned future work will include an in-depth study on each recovery stage to ascertain recovery kinetics and extract migration energies. Furthermore, time-resolved temperature dependent photoluminescence studies are being performed in an attempt to correlate with the resistivity measurements. Currently, we are working on incorporating this method in the study of other semiconductor films and intend to expand the research further by implementing it for the study of self-irradiating effects in metal oxides.

## Methods

### Materials and Chemicals

Sodium hydroxide (Gadot, AR), lead (Pb^2+^) nitrate (Aldrich, analytical 99.99+%), thiourea (Aldrich, ACS ≥99.0%), and tetravalent thorium cations (Aldrich, 1000 ppm of ^232^Th^4+^ in 5.1 wt% HNO_3_) were used without further purification for deposition of PbS. Single crystal GaAs(100) substrates were purchased from AXT (epi-polished, undoped, ±0.1° miscut). The substrates were cleaved into 1 × 1.5 cm^2^ rectangles and cleaned with distilled water, then with analytical ethanol and dried. Distilled water was obtained using a Millipore Direct Q3. Radioactive ^228^Th was purchased from Eckert-Ziegler and currently aged at ~2.5 years. Original activity was 1 mCi in 0.5 ml of 1 M nitric acid.

The deposition procedure has been described in detail by Biton *et al*.^21^ and Osherov *et al*.^42^. In order to accomplish deposition using minute amounts of solution, concentrations were kept the same as in ref. 21, but the solution volumes were minimized to a final solution volume of 2 ml. The substrates were placed epi-side down in the solution, at an angle of ca. 80° with respect to the air–solution interface.

### Structural and chemical characterization

#### X-Ray Diffraction (XRD)

The crystallographic phase and the orientation of the films were studied by XRD. A Panalytical Empyrean powder diffractometer equipped with PIXcel linear detector and monochromator on diffracted beam was used. Data were collected in the 2θ/θ geometry using Cu Kα radiation (λ = 1.5405 Å) at 40 kV and 30 mA. Diffraction patterns were taken during 8 minutes in a 2θ range of 20–65° with a step size of ~0.039°.

#### Field Emission Gun Scanning Electron Microscope (FEG-SEM)

The morphology of the films was observed using an ultrahigh resolution JEOL JSM-7400F FEG-SEM without coating of the surface. The acceleration voltage ranged from 1 to 5 kV. Film thickness was measured from cross sections while surface topography was observed in plan-view.

### Sample radiative emission characterization

#### Gamma spectroscopy

The total activity of ^228^Th cannot be measured directly using γ-spectroscopy, due to its negligible gamma emission. However, when ^228^Th is in equilibrium with its daughters (^224^Ra and ^212^Pb), its activity can be determined through that of the daughters. Gamma ray measurements were carried out in a well-type 2″ diameter NaI detector (1282 CompuGamma, LKB Wallac).

#### Alpha spectroscopy

Measuring the alpha spectrum of the ^228^Th decay chain in the sample provides meaningful experimental understanding regarding the properties of the film. The overall count of the ^228^Th alpha line gives directly the total activity of ^228^Th. In addition, the energy of this line (as well as daughter isotopes in the spectrum) provides an estimate of the average depth of the decaying isotope in the sample, which is a consequence of the fact that alpha particles emitted from deep in the sample, lose some energy on the way out^43, 44^. Furthermore, the fact that the alpha lines corresponding to isotopes down the chain are not in equilibrium with that of ^228^Th implies that some of them actually desorb out of the sample. The probability of this desorption effect is related to the average depth of the ^228^Th distribution in the sample. The α-spectroscopy measurements were performed on a 150 mm^2^ ion-implanted Si detector (EG&G ORTEC), using a multi-channel analyzer with a sensitivity of 2.8 keV/channel.

#### FUJI autoradiography

Fuji autoradiography is a technique which allows measurement of the amount of energy deposited by ionizing radiation at any point in the film. Thus, by exposing the radioactive sample to the FUJI film in contact, the distribution of the alpha activity (which is the dominant source of deposited energy) on the film can be determined. Analyzing the FUJI film was done on a Fujifilm FLA-9000 instrument, with a lateral resolution range of 10 to 200 micrometers.

#### Daughter desorption probability

During the α-decay of ^228^Th, the daughter nucleus ^224^Ra recoils with energy of about 100 keV. This energy is enough to traverse about 20 nm in the PbS film. Thus, ^224^Ra atoms originating from a depth less than 20 nm have a finite probability of desorbing from the sample surface. The desorption probability of the sample is defined as the ratio of desorbed atoms to the total amount of created ^224^Ra atoms. This probability is determined by collecting the desorbed atoms on a test foil and measuring their total activity, taking into account the temporal sequence of the procedure. This desorption probability is related to the average depth of the ^228^Th assuming a homogeneous depth distribution of the parent isotope.

### Electrical resistivity measurements

The resistivity of the samples was measured using the four probe resistivity technique. A four probe JANIS Research model CCR12-2-2HMF station was used combined with a Stanford Research SR830 lock-in amplifier. An alternating voltage (50 mV @ 15 Hz) combined with an external shunt resistor of 10 MΩ was used to accurately determine the current flow. The samples were cooled by a cryogenic cold-head system using closed loop helium gas, based on the Gifford-McMahon thermodynamic cycle. The sample temperature was controlled by a 50 Ω Ni-Cr heater and measured by four calibrated silicon diodes.

Prior to damage accumulation measurements sample resistivity was determined as a function of temperature. This was done both to establish a base value and to ascertain repeatability when the sample is cooled back and forth in annealing measurements. To accumulate damage samples were kept at the base temperature of 12 °K while monitoring the change in resistivity. The samples were heated to a specific annealing temperature, were kept for 10 min and then cooled back to the original base temperature at which the modified resistivity was measured. This procedure was repeated for a set of monotonically increasing temperatures.

## Electronic supplementary material

supplementary information
