# Supplementary material for: A New Solid Solution Approach for the Study of Self-Irradiating Damage in non-Radioactive Materials
Source: Sci Rep. 2017 Jun 5;7:2780. doi: 10.1038/s41598-017-03150-9 (PMC5459804; doi:10.1038/s41598-017-03150-9)
Supplement: Supplementary file 1 — supplementary information [file 41598_2017_3150_MOESM1_ESM.pdf]

Supporting Information

A New Solid Solution Approach for the Study of Self-Irradiating Damage in non-Radioactive Materials

*Tzvi Templeman[1,2], Michael Shandalov[3], Michael Schmidt[4], Amir Tal[2,5], Gabby Sarusi[2,5], Eyal Yahel[3], Itzhak Kelson[4] and Yuval Golan[1,2]**

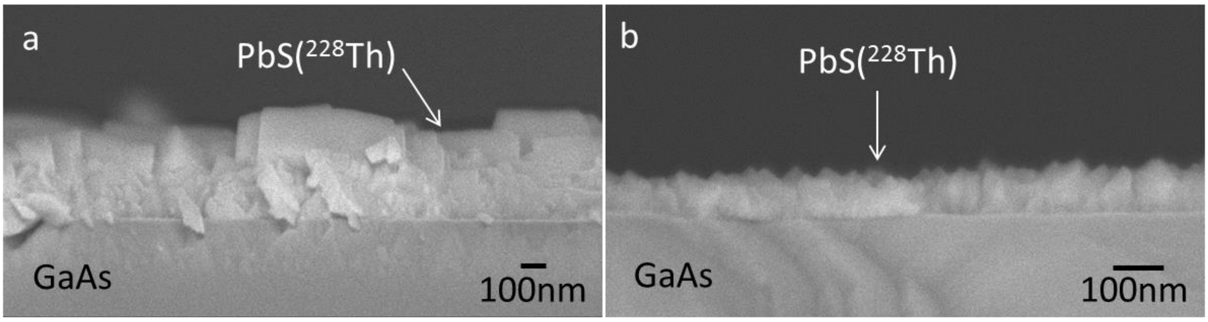

Figure S1. SEM cross section imaging of PbS(^{228}Th) thin films grown at (a) pH 13.3 and (b) pH12.8.

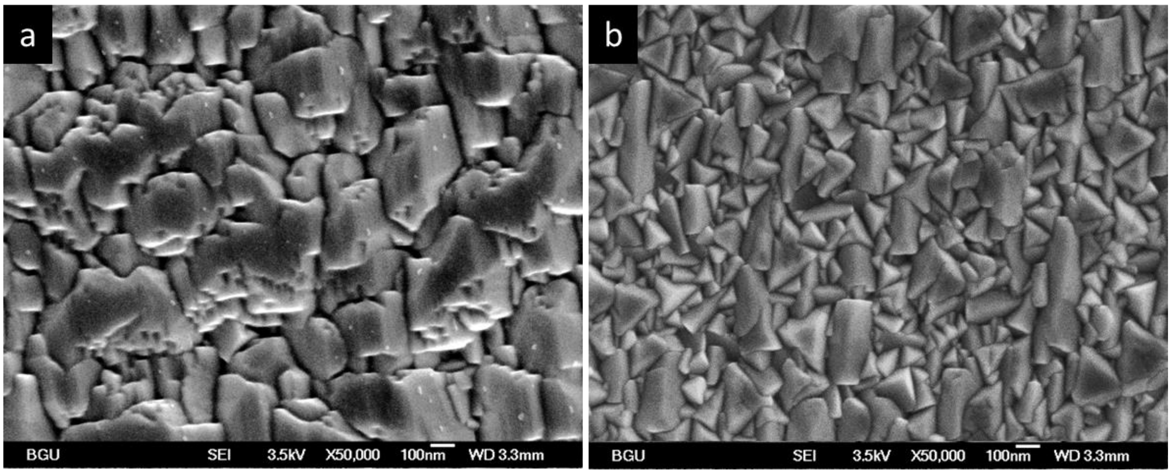

Figure S2. SEM plan view imaging of (a) PbS(0.15ppm ^{228}Th) and (b) PbS(0.15at% ^{232}Th) thin films.

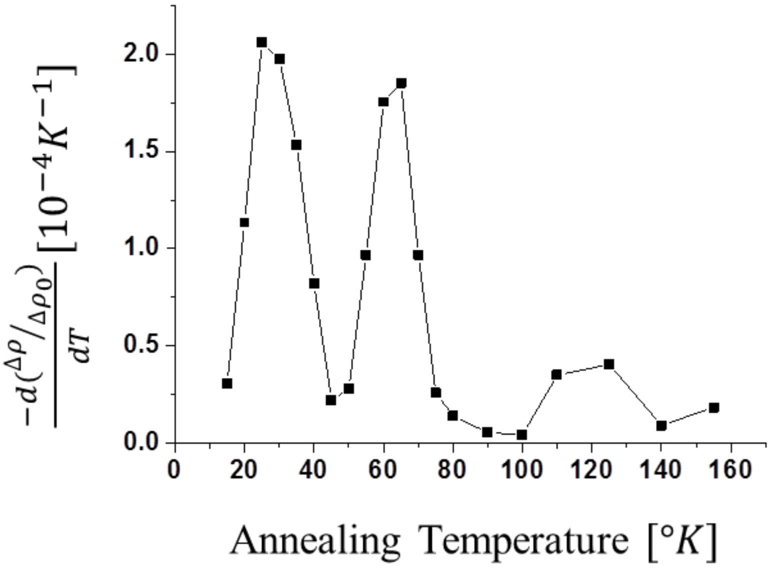

Figure S3. Derivative with respect to temperature of resistivity recovery (Figure 6a). This method is implemented to accurately assess step temperature (Table 2).
